# Impact of socio-economic factors on stroke prevalence among urban and rural residents in Mainland China

**DOI:** 10.1186/1471-2458-8-170

**Published:** 2008-05-21

**Authors:** Fei Xu, Lap Ah Tse, XiaoMei Yin, Ignatius Tak-sun Yu, Sian Griffiths

**Affiliations:** 1 Nanjing Municipal Center for Disease Control & Prevention, Nanjing, PR China; 2 Department of Community and Family Medicine, The Chinese University of Hong Kong, Hong Kong, PR China; 3 Center for Occupational and Environmental Health Studies, School of Public Health, The Chinese University of Hong Kong, PR China; 4 School of Public Health, The Chinese University of Hong Kong, PR China

## Abstract

**Background:**

An inverse relationship between better socioeconomic status (total household income, education or occupation) and stroke has been established in developed communities, but family size has generally not been considered in the use of socioeconomic status indices. We explored the utility of Family Average Income (FAI) as a single index of socioeconomic status to examine the association with stroke prevalence in a region of China, and we also compared its performance as a single index of socioeconomic status with that of education and occupation.

**Methods:**

A population-based cross-sectional study was conducted in Nanjing municipality of China during the period between October 2000 and March 2001. A total of 45 administrative villages were randomly selected using a multi-stage sampling approach and all regular local residents aged 35 years or above were included. Descriptive statistics and logistic regression models were used in analysis.

**Results:**

The overall prevalence of diagnosed stroke was 1.54% in all 29,340 eligible participants. An elevated prevalence of stroke was associated with increasing levels of FAI. After adjustment for basic demographic variables (age, urban/rural area and gender) and a group of defined conventional risk factors, this gradient still remained significant, with participants in the highest (OR = 1.94, 95% CI = 1.40, 2.70) and middle (OR = 1.43, 95% CI = 1.01, 2.02) categories of FAI having higher risks compared with the lowest category. A significantly elevated OR of stroke prevalence was found in white collar workers compared to blue collar workers, while no significant relationship was observed with education.

**Conclusion:**

Our study consistently revealed that the prevalence of stroke was associated with increasing levels of all SES indices, including FAI, education, and occupation. However, a significant gradient was only observed with FAI after controlling for important confounding factors. The findings suggested that, compared with occupation and education, FAI could be used as a more sensitive index of socio-economic status for public health studies in China.

## Background

Previous studies using different study designs and various definitions of socioeconomic status (SES) have shown that people with lower SES are at increased risk of stroke [1-6]. Such socioeconomic gradient for the risk of stroke tends to be flattened, though still significant, after taking into account the conventional risk factors of stroke, including cigarette smoking, alcohol drinking, body mass index (BMI), physical activity, diabetes, and hypertension [6-12].

SES is a complex construct containing either a single measure or a combination of several components. Using different SES measures may reflect different specific exposures and can provide various information on peoples' relative socioeconomic positions within their community [13]. It has been suggested that family average income (FAI) could be considered as a single measure of SES as it is a more realistic reflection of material measures than other indicators such as educational attainment or occupation [14-16].

In our previous studies, we found that overweight and diabetes, two conventional risk factors of stroke, [17-19] were positively associated with FAI in China [15,16]. We thus hypothesized that Chinese people with higher FAI might also be at an increased risk of stroke. The purpose of this study was to examine the relationship between stroke and SES, indicated separately by FAI, education and occupation, and to see if the SES gradient, if present, could be explained by differences in conventional risk factors of stroke, using representative data obtained through a large population-based survey conducted in both urban and rural areas of a region in Mainland China between October 2000 and March 2001.

## Methods

### Sample selection and participants

This is a large-scale population-based cross-sectional study (Nanjing Chronic Disease and Risk Behaviour Survey, NCDRBS) conducted in Nanjing municipality. Nanjing, the capital of Jiangsu province, is located in the east of China and has a population of more than 5.6 million in 2000. In Mainland China, the administrative system consists of 5 strata: Central government, Provincial/Municipal government, District/County government, Street/Town Government and Administrative Village. It has 15 administrative units, ten being urban and five rural. A multi-stage random sampling method was adopted. First, we randomly selected three urban districts and two rural counties. This was followed by randomly selecting three streets/towns from each chosen district/county. Finally, three administrative villages in each street/town were randomly selected. This resulted in a total number of 45 villages.

According to a previous study conducted in the local province, the overall self-reported prevalence of stroke was 2.6% among people aged 15 years old or above [20]. Considering the prevalence of stroke diagnosed by medical doctors might be lower than the overall prevalence, a conservative prevalence of diagnosed stroke of 1.0% of this study was assumed, and about 320 stroke cases were expected from this representative sample. To explore the association of stroke prevalence with SES indices of our study, about 300 prevalent stroke cases and an equal number of subjects without stroke were required to detect a statistically significant (p < 0.05) odds ratio (OR) of 1.5 assuming the statistical power of 80%. The statistical power would be increased given more non-stroke subjects being obtained based on the scenario of cross-sectional study design.

There is a residence registration system in Mainland China. All Chinese citizens are regulated to register their basic demographic information (birth date, sex, residence) in a department of local government (usually the Street/Town Government). Those registered residents are called local permanent residents, and their information is included in the authorities' database of census. Thus, the local authority has the demographic information of all permanent residents registered in each administrative area. This unique administrative system allows us to collect the basic demographic information on all permanent residents registered in all selected administrative villages, including both responders and non-responders, and also allows us to make the comparison between responders and non-responders. One month before the household interview, government administrators of the selected streets, towns and administrative villages were informed of the study. With their consents and supports, we advertised our program via local TV broadcasting, and distributed flyers to each household. All households of the selected villages were invited to participate in the study. Informed personal and family oral consent was obtained before each household interview. To be eligible for inclusion in the study, participants had to be aged 35 years or above and to have been a local resident for at least 5 years.

This survey was granted approval by the academic and ethical committee of Nanjing Municipal Center for Disease Control and Prevention in accordance with internationally agreed ethical principles for the conduct of medical research.

### Questionnaire

After informed consent was obtained, all eligible subjects of each household were asked to complete a standardized questionnaire administered by the trained interviewers (healthcare professionals) who were trained in standard interview procedures prior to survey administration. The household was based on the family unit in this study. Information collected in the structured questionnaire included age, gender, area of residence, stroke status, number of family members, total monthly income of family, educational attainment, occupation, diabetic status, smoking status, alcohol drinking, and physical activity. Blood pressure, body weight and height were measured at local clinics using calibrated equipment.

### Definitions

A family was defined as those who lived together and shared living-related expenditures. The total monthly income of all family members is the monthly total earnings of the whole family, including salaries, pensions and allowances, money from selling goods and products, and the estimated market-price value of home grown products (including rice, wheat, vegetables, fruits and milk) for personal consumption. Family average income (FAI) was defined as the total monthly incomes of all family members divided by the total number of all family members (including children and elderly), and FAI was categorized into three levels by tertiles: lower, middle and higher [15].

### Outcomes

Participants were asked to respond to the question "Have you ever been diagnosed as a stroke patient by a doctor or doctors at a grade 1 and higher hospital?" If the answer was "yes", the participant would be asked to show his/her medical records to confirm their declaration. Details about sub-types of stroke, date of diagnosis, hospital of diagnosis, evidence related to the diagnosis, and the current medication status were extracted from the medical records kept by the patients themselves or their family members. Thus, all self-reported stroke patients or families with stroke patents supplied medical records to confirm their declarations. For those patients who were unable to answer questions due to post-stroke functional disabilities or dementia, all the questions were answered by the relatives in the same family. No medical records were traced if the subjects answered "No" for a diagnosis of stroke. We assumed that no participants or their family members would forget/ignore experiencing a stroke event.

In China, the WHO MONICA criteria were used to make the diagnosis of stroke in hospitals [21]. We did not take into consideration the sub-types of stroke, but just classified the stroke status as non-stroke and stroke as the outcome variable in our analysis. Conventional stroke risk factors considered in this study included BMI, cigarette smoking, alcohol consumption, diabetes, high blood pressure, as well as occupational and leisure time physical activities. All these conventional risk factors were defined and categorized elsewhere [15].

### Data management and analysis

The associations between stroke and FAI categories, education or occupation were examined using the chi-squared test. To control for potential confounders, multiple logistic regression analyses were used to estimate the adjusted odds ratios and the 95% confidence intervals (95% CIs) of having stroke after the adjustment of the conventional stroke risk factors based on the following three models. Model 1: only FAI/education/occupation was as the single predictor; Model 2: age, gender and residence area were added to Model 1; Model 3: a group of defined conventional risk factors of stroke, including BMI, smoking status, alcohol drinking, diabetes, high blood pressure, occupational and leisure-time physical activity were further introduced to the Model 2.

Data were double-entered and cleaned with Epi Info (Version 6.04, 2002; CDC, Atlanta, Georgia, USA), and managed and analyzed using SPSS (Version 11.0.1, 2001; SPSS Chicago, Illinois, USA). Data were weighted according to sample scheme coefficients.

## Results

Among a total of 15,563 families and 32,568 eligible subjects approached in this survey, 15,300 households and 29,340 subjects agreed to participate in the interview, giving a response rate of 98.3% (households) and 90.1% (individual participants) respectively. The average family size was 3.3 members (SD = 1.3, Min = 1 and Max = 9).

No significant differences were detected between the respondents and the non-respondents in terms of age, gender and residence area. No family had more than one stroke patients at the time of interview. There were 67.7% of participants from urban areas and 32.3% from rural areas; 49.8% of subjects were male and 50.2% were female.

Table 1 shows the relationships between the FAI and a group of defined conventional risk factors of stroke in this study. Except for gender, all other risk factors were significantly associated with FAI. Higher levels of FAI were observed with increasing levels of education, and BMI, as well as with high blood pressure, diabetes, and living in the urban area. Higher proportions of smokers, alcohol drinkers, and taking physical activities were observed in the lower categories of FAI.

**Table 1:** Association of family average income and conventional potential confounders

Characteristic	Family Average Income (n, %*)			p value**
		
	Lower (N = 9689)	Middle (N = 8991)	Higher (N = 10660)	
**Age group(yrs)**				
35–49	4127 (42.6)	4880 (54.3)	4205 (39.4)	< 0.001
50–64	3142 (32.4)	2696 (30.0)	4115 (38.6)	
65+	2420 (25.0)	1415 (15.7)	2340 (22.0)	
**Gender**				
Female	5063 (52.3)	4436 (49.3)	5243 (49.2)	0.581
Male	4626 (47.7)	4555 (50.7)	5417 (50.8)	
**Area**				
Rural	6469 (66.8)	2624 (29.2)	386 (3.6)	< 0.001
Urban	3220 (33.2)	6367 (70.8)	10274 (96.4)	
**Education (yrs)**				
0–9	9018 (93.1)	6872 (76.4)	3947 (37.0)	< 0.001
10–12	641 (6.6)	1770 (19.7)	3070 (28.8)	
13+	30 (0.3)	349 (3.9)	3643 (34.2)	
**Occupation**†				
Blue collar	7585 (78.3)	5538 (61.6)	2742 (25.7)	< 0.001
Service people	1681 (17.3)	1326 (14.7)	652 (6.1)	
White collar	423 (4.4)	2127 (23.7)	7266 (68.2)	
**BMI (kg/m2)**				
24-	6761 (69.8)	5366 (59.7)	5967 (56.0)	< 0.001
24–28	2362 (24.4)	2834 (31.5)	3757 (35.2)	
28+	566 (5.8)	791 (8.8)	936 (8.8)	
**Cigarette Smoking**				
Never	6393 (66.0)	6060 (67.4)	7994 (75.0)	< 0.001
Ex/Current	3296 (34.0)	2931 (32.6)	2666 (25.0)	
**Alcohol consumption**				
No	7468 (77.1)	7099 (79.0)	9003 (84.5)	< 0.001
Ex/Current	2221 (22.9)	1892 (21.0)	1657 (15.5)	
**Diabetes**				
No	9640 (99.5)	8844 (98.4)	10227 (95.9)	< 0.001
Yes	49 (0.5)	147 (1.6)	433 (4.1)	
**High blood pressure**				
No	6505 (67.1)	6108 (67.9)	6987 (65.5)	0.013
Yes	3184 (32.9)	2883 (32.1)	3673 (34.5)	
**Occupational physical activity**				
Light	4292 (44.3)	6242 (69.4)	9761 (91.6)	< 0.001
Moderate	1125 (11.6)	1157 (12.9)	643 (6.0)	
Vigorous	4272 (44.1)	1592 (17.7)	256 (2.4)	
**Leisure-time physical activity**				
Light	7281 (75.1)	7502 (83.4)	8957 (84.0)	< 0.001
Moderate	2089 (21.6)	1374 (15.3)	1643 (15.4)	
Vigorous	319 (3.3)	115 (1.3)	60 (0.6)	

* Percentages across column.

**P value between sub-groups of each variable.

†Blue collar = farmer, factory worker, forestry worker, fisher and military person; Service people = salespeople, house worker and vehicle driver; White collar = office worker, teacher, doctor and academic researcher, government officer.

Table 2 presents the associations of diagnosed stroke with age, gender and area of residence. The overall prevalence of diagnosed stroke was 1.54% for participants aged 35 years or above. By holding the group of defined conventional risk factors constant, the prevalence of stroke in participants aged 65 years or above was 41.8 times as high as that in people aged 35–49 years (95%CI = 25.3, 69.2). The OR for stroke among subjects living in the urban area was 2.78 (95%CI = 1.86, 4.16) compared with their rural counterparts. Men had higher OR of stroke than women (adjusted OR = 1.56, 95%CI = 1.25, 1.94).

**Table 2:** Prevalence of stroke (% and n/N) by age, gender and urban-rural residence in Nanjing, China

	Stroke n (%)	Non-stroke n (%)	Adj. odds ratio (95% CI) *
Age-group			
35–44	16 (0.12)	13196 (99.88)	1
45–64	139 (1.40)	9814 (98.60)	7.42 (4.40, 12.54)
65+	298 (4.83)	5877(95.17)	22.39 (13.38, 37.48)
Area			
Rural	37 (0.39)	9442 (99.61)	1
Urban	416 (2.09)	19455 (97.91)	2.78 (1.86, 4.16)
Gender			
Female	186 (1.26)	14556 (98.74)	1
Male	267 (1.83)	14331 (98.17)	1.56 (1.25, 1.94)

n = number of participants with stroke within sub-group; N = total number of participants within sub-group.

* Odds ratios adjusted for Odds ratios adjusted for BMI, smoking status, alcohol drinking, diabetes, high blood pressure, occupational and leisure-time physical activity.

Table 3 compares the associations of stroke prevalence with each indicator of SES using three different models. Univariate logistic regression analyses from Model 1 showed that increased risks of stroke prevalence were strongly associated with the increasing level of FAI. A significantly higher OR was found in the white-collar workers (OR = 5.83, 95% CI = 4.62, 7.35) and the service workers (OR = 1.59, 95% CI = 1.07, 2.36) compared to the blue-collar workers. Only the participants receiving the highest education showed a significantly increased OR of stroke compared with the lowest category (OR = 2.12, 95% CI = 1.70, 2.65). Model 2, which was adjusted for age, gender, area of residence, showed that all the associations obtained from model 1 were attenuated but remained statistically significant, with the exception of service workers (adjusted OR = 0.98). The gradient relationships with stroke prevalence were flattened after a group of defined conventional stroke risk factors were further introduced into the model (Model 3). The association between education and stroke became non-significant.

**Table 3:** Prevalence of stroke (% and n/N) and its association with Family Average Income (FAI), Education and Occupation in Nanjing, China

	Prevalence (n and %)		Adj. odds ratio (95% CI)		
	
	Stroke	Non-stroke	Model 1*	Model 2 ^§^	Model 3^#^
**Overall**	453 (1.5)	28887 (98.5)			
**FAI (Tertile)**					
Lower	63 (0.7)	9626 (99.3)	1	1	1
Middle	107 (1.2)	8884 (98.8)	1.84(1.35, 2.52)	1.59(1.13, 2.23)	1.43(1.01, 2.02)
Higher	283 (2.7)	10377 (97.3)	4.17 (3.17, 5.48)	2.32(1.69, 3.19)	1.94(1.40, 2.70)
**Education (yrs)**					
0–9	269 (1.4)	19568 (98.6)	1	1	1
10–12	70 (1.3)	5411 (98.7)	0.94 (0.72, 1.23)	1.18(0.89, 1.55)	1.08(0.82, 1.44)
13+	114 (2.8)	3908 (97.2)	2.12 (1.70, 2.65)	1.36(1.07, 1.72)	1.18(0.92, 1.51)
**Occupation†**					
Blue collar	93 (0.6)	15772 (99.4)	1	1	1
Service	34 (0.9)	3625 (99.1)	1.59 (1.07, 2.36)	0.89(0.60, 1.34)	0.80(0.53, 1.21)
White collar	326 (3.3)	9490 (96.7)	5.83 (4.62, 7.35)	1.98(1.52, 2.57)	1.64(1.25, 2.15)

n = number of participants with stroke within sub-group; N = total number of participants within sub-group. The lower FAI was the reference category.

* Model 1: Only FAI, education or occupation was entered logistic regression model as the single predictor, the odds ratios were un-adjusted.

© Model 2: Odds ratios adjusted for age, gender, area of residence.

# Model 3: Odds ratios adjusted for age, gender, area of residence, BMI, smoking status, alcohol drinking, diabetes, high blood pressure, occupational and leisure-time physical activity.

†Blue collar = farmer, factory worker, forestry worker, fisher; Service people = salespeople, house worker; White collar = office worker, teacher, doctor.

Table 4 showed that the prevalence of stroke increased from the lowest to the highest FAI category among people aged 50 years or above, living in urban area, all education categories, blue collar workers, and in both genders. Positive and significant associations between FAI and stroke prevalence were observed among the elderly, males, urban area, lowest education category, and the service workers, after the multiple logistic regression analyses were performed, while the numbers of observations were small and the confidence intervals were relatively wide in some categories.

**Table 4:** Prevalence of stroke (% and n/N) and its association with Family Average Income (FAI) by basic demographic variables in Nanjing, China

Variables	FAI Category	Stroke (% and n)	Non-stroke (% and n)	Unjusted Odds ratio (95%CI)	Adjusted Odds ratio† (95%CI)
**Age (yrs)**					
35–49					
	Lower	8 (0.19)	4119 (99.81)	1	1
	Middle	6 (0.12)	4874 (99.88)	0.94(0.39, 2.31)	0.33(0.11, 1.00)
	Higher	2 (0.05)	4203 (99.95)	0.94(0.39, 2.31)	0.08(0.02, 0.49)
50–64					
	Lower	22 (0.70)	3120 (99.30)	1	1
	Middle	37 (1.37)	2659 (98.63)	2.37(1.24, 4.50)	0.91(0.51, 1.63)
	Higher	80 (1.94)	4035 (98.06)	3.50(1.81, 6.78)	0.99(0.56, 1.75)
65+					
	Lower	33 (1.36)	2387 (98.64)	1	1
	Middle	64 (4.52)	1351 (95.48)	3.51(1.79, 6.89)	1.96(1.23, 3.13)
	Higher	210 (8.59)	2139 (91.41)	5.21(2.63, 10.32)	3.21(2.06, 4.99)
**Gender**					
Women					
	Lower	25 (0.49)	5038 (99.51)	1	1
	Middle	43 (0.97)	4393 (99.03)	2.43(1.23, 4.79)	1.28(0.81, 2.02)
	Higher	118 (2.25)	5125 (97.75)	3.73(1.88, 7.40)	1.51(0.98, 2.33)
Men					
	Lower	38 (0.82)	4588 (99.18)	1	1
	Middle	64 (1.41)	4491 (98.59)	2.36(1.40, 3.97)	1.59(0.92, 2.72)
	Higher	165 (3. 50)	5252 (96.50)	3.85(2.26, 6.58)	2.64(1.58, 4.40)
**Area**					
Rural					
	Lower	28 (0.43)	6441 (99.57)	1	1
	Middle	8 (0.30)	2616 (99.70)	1.38(0.54, 3.48)	0.93(0.41, 2.12)
	Higher	1 (0.26)	387 (99.74)	1.23(0.16, 9.58)	0.59(0.08, 4.54)
Urban					
	Lower	35 (1.09)	3185 (98.91)	1	1
	Middle	99 (1.55)	6268 (98.45)	2.92(1.77, 4.82)	1.58(1.06, 2.34)
	Higher	282 (2.74)	9992 (97.26)	4.64(2.82, 7.64)	2.63(1.95, 3.55)
**Education (yrs)**					
0–9					
	Lower	59 (0.65)	8959 (99.35)	1	1
	Middle	90 (1.31)	6782 (98.69)	2.02(1.45, 2.80)	1.46(1.01, 2.10)
	Higher	120 (3.04)	3827 (96.96)	4.76 (3.48, 6.52)	2.01(1.39, 2.90)
10–12					
	Lower	4 (0.62)	637 (99.38)	1	1
	Middle	15 (0.85)	1755 (99.15)	1.36(0.45, 4.12)	0.63(0.19, 2.07)
	Higher	51 (1.66)	3019 (98.34)	2.69 (0.97, 7.47)	0.65(0.21, 2.01)
13+					
	Lower	0 (0.00)	30 (100.0)	1	1
	Middle	2 (0.57)	347 (99.43)	N/A	N/A
	Higher	112 (3.07)	3531 (96.93)	N/A	N/A
Occupation*					
Blue collar					
	Lower	37 (0.49)	7548 (99.51)	1	1
	Middle	36 (0.65)	5502 (99.35)	1.34(0.84, 2.12)	1.33(0.80, 2.21)
	Higher	20 (0.73)	2720 (99.27)	1.50 (0.87, 2.59)	1.11(0.59, 2.07)
Service people					
	Lower	13 (0.77)	1668 (99.23)	1	1
	Middle	10 (0.75)	1316 (99.25)	0.98(0.43, 2.23)	1.43(0.58, 3.51)
	Higher	11 (1.69)	641 (98.31)	2.20(0.98, 4.94)	3.49(1.32, 9.24)
White collar					
	Lower	13 (3.07)	410 (96.93)	1	1
	Middle	61 (2.87)	2066 (97.13)	0.93(0.51, 1.71)	0.91(0.49, 1.70)
	Higher	252 (3.47)	7014 (96.53)	1.13(0.64, 2.00)	1.18(0.66, 2.13)

n = number of participants with stroke within sub-group; N = total number of participants within sub-group.

†Odds ratios adjusted for age, gender, area of residence, BMI category, smoking status, alcohol drinking, diabetes, high blood pressure, occupational and leisure-time physical activity.

N/A = not applicable.

*Blue collar = farmer, factory worker, forestry worker, fisher; Service people = salespeople, house worker; White collar = office worker, teacher, doctor and retired people.

Lower FAI = the reference category.

## Discussion

Our study consistently revealed that the risk of stroke was associated with increasing levels of all SES indices, including FAI, education, and occupation. A positive and significant gradient of FAI with stroke prevalence remained after the adjustment of the basic demographic variables (age, gender and area) and a group of defined conventional risk factors. The findings of our study show similar patterns to those of previous decades for Western populations but were in contrast to the results in more recent developed areas in which SES is inversely associated with the risk of stroke [1-12]. We hypothesize that this reflects the earlier stage of epidemiological transition within China at this time, in which more affluent areas are adopting more Westernized lifestyles including diet.

Socioeconomic inequalities in health have been attributed to a number of different mechanisms, including unhealthy behaviors, inadequate accesses to health care service, nutritional inadequacies, other inequalities in material circumstances, and psychosocial stress [22-24]. The mechanism of how the specific SES influences stroke is not entirely understood. Potential explanations for the patterns of inequalities may be related to the differences in major risk factors of stroke, in psychosocial factors, or in access to and use of health care services [1,5], since in China ability to pay is linked to access to health care.

However, psychosocial factors and inequalities of health care did not adequately explain the SES gradient in stroke, because the social gradient in stroke has been shown to be largely driven by conventional stroke risk factors [12]. Previous studies suggested that the conventional risk factors (components were similar to our study) might have accounted for more than half of stroke disparities [6,25]. It is well known that, in developing countries, people in lower SES categories are generally less likely to be obese and more likely to be physically active in both their workplace and leisure time than their counterparts in developed countries [26-32]. People with lower socioeconomic status may also have a lower total food (calorie) intake, especially of animal proteins; they are more likely to consume vegetables [29-32]. As seen in many areas of Africa, people of low SES status were less likely to be overweight and develop diabetes due to the nature of their work and also access to transportation or car ownership [15,16]. Our study found that the proportion of subjects who consumed red meat at least once a day was significantly higher in the high FAI group than the low FAI category (87.8% vs. 46.0%, p < 0.001), while the rate of participants who consumed vegetables at least 100 gram each day in the higher FAI group was significantly lower than those in the lower FAI group (59.6% *vs*. 72.4%, p < 0.001). These disparities in conventional risk factors might act as intermediates for the impact of SES on stroke development. However, the proportion of cigarette smokers among the lower FAI group was significantly higher than that in the higher FAI group (37.4% vs. 29.1%, p < 0.01). Our finding on the negative association between the prevalence of cigarette smoking and SES level was inconsistent with the evidence from previous studies that smoking was an important factor explaining stroke disparities in the different socioeconomic groups [6-9]. However, no firm conclusion could be drawn based on the current cross-sectional study design. It would be of great interest to analyse the trend of stroke prevalence and its association with SES in the context of China to see whether and when the relationship between stroke and SES would reverse and become similar to that observed in developed countries.

We believe that per capita income of each family unit was probably a more sensitive reflection of the influences of SES on participants' lifestyles than per capita GDP of the surveyed area. Compared to other SES indicators (such as educational attainment, occupation, total household income or deprivation score), FAI in China was recognized as a new and a single measure of SES based on our findings since it was considered to be a more realistic reflection of material measures. In China, most families have only one child, but meanwhile a lot of families have their parents and/or grandparents living together, especially in the rural areas. The family size is mostly affected by the number of supported elderly. Most supported elderly may have lower retirement salaries or even no incomes (rural elders) at all. Total household incomes can thus not reflect per capita incomes in the families. With the same household incomes, a larger family with more elderly living together may have poorer life quality than that of a smaller family due to increased costs of supporting a larger family. Although education and occupation may partially determine an adult's SES in Mainland China where the economy is undergoing a rapid transition, they were considered to be less valid and sensitive than FAI and could only act as an indirect index of SES in the context of China.

An important question is whether competing causes of death might play an important role in the explanation of the inverse association between FAI and stroke prevalence. It was possible that subjects in the low income groups could have died from other causes such as infectious diseases in earlier life that prevented them from living long enough to develop stroke later in life. Vital statistics showed that the ranking of mortality from specific diseases among urban population was similar to that of the rural areas. In addition, the life expectancy was over 73 years in the rural areas of Nanjing since 1995. Thus, we believe that the lower prevalence of stroke among the lower SES groups could not be adequately explained by competing causes of deaths in earlier lives.

Elevated OR of stroke was significantly associated with higher SES. However, this gradient pattern remained significant only for the age group of 65 years or above after stratification by age. This might be due to the low prevalence of stroke among the younger (0.12%) and middle age groups (1.40%). The prevalence tended to be unstable after the stratification by FAI (three categories). Another possible explanation was that the relationship between SES and stroke has been transiting to that observed in Western countries, because younger people adopted more westernised lifestyles (e.g. high fat intakes) relative to their old counterparts.

The strengths of the current study is that the analysis include a large representative sample of both urban and rural populations, a very high response rate of more than 90% for household interviews, a sensitive and realistic index of SES (the FAI), and a full consideration of important stroke risk factors. However, when interpreting these findings, several limitations of this study should be considered. First, although strong agreement was demonstrated between self-reported stroke cases and those confirmed by medical record review or CT scan [19,34,35], the possibility of underestimating the prevalence of diagnosed stroke could not be completely ruled out in our study, especially in rural areas. However, in the current context of healthcare in China, all outpatients can keep their personal medical records and/or hospital discharge notes from which we were able to check and extract the information regarding the dates of hospital visits, diagnoses of diseases, prescriptions, treatment, and recommendations. We believe most people knew well their disease status, especially for strokes requiring hospitalization with certain recognized disabilities. Based on this scenario, we believe that the possibility of underreporting diagnosed strokes by patients themselves, if present, would be minimal. The second limitation was related to self-reported family incomes. People living in rural areas might have underestimated their earnings and inaccurately estimated the value of their home-grown foods and informal barter arrangements. We consider such misclassification was likely to be random and biased the associations towards null, as subjects with stroke and those without stroke should not have estimated their incomes differently. Third, no data was obtained on participants' accesses to health care, which makes it difficult to understand the role of inequitable access to health care on stroke prevalence. The prevalence of stroke was affected by both the incidence and duration of survival. We found that people living in urban areas had a higher prevalence of stroke regardless of the SES levels (Table 4). More accessible and affordable health care for urban people might result in a better post-stroke survival and could partly explain the disparities of stroke prevalence between urban and rural populations. Similarly, people with higher FAI might have better access to medical facilities and better post stroke health care, which could contribute to extending their life expectancies beyond that of those with lower FAI status. The positive gradient between stroke prevalence and FAI might be influenced by the survivor effect, as prevalent cases instead of incident cases were studied with the current cross-sectional design. Fourth, our study did not allow us to infer causality for the stroke-FAI relationships. As the study was cross-sectional, the temporal relationship between SES and stroke could not be accurately defined. Part of their association might reflect the impact of stroke on the FAI due to the loss of work time of both the stroke patients and the family members. A prospective study design to confirm the potential causal relationship between family average income and stroke is recommended. Finally, this study was mainly designed to investigate the prevalence of selected chronic diseases in Nanjing municipality, thus only each responder's identification number was originally required to be recorded on each questionnaire without consideration of family identification number. At this time, several years after household interview, it was impossible for us to check up each responder's family identification by manual counting the local residence registration information. This inherent limitation makes us unable to control for possible clustering effect in the analyses.

We recommend conducting further in-depth studies to elucidate the relationships between stroke (including its conventional risk factors) and SES under different circumstances in countries undergoing demographic and epidemiological transitions. In a developing society like China with rapid social and economic transition to industrialization, more and more people are being employed as office workers and their lifestyles become westernized (e.g., consuming foods with high energy and high fat but taking less physical activity and having less Chinese traditional vegetable and rice foods) [36]. As a consequence of 'unhealthy' lifestyles, more and more people are tending to become overweight, especially in urban areas [36]. Health promotion strategies and lifestyle interventions targeting people with higher family average income in the current context of China could be helpful in the campaigns against stroke. Meanwhile, to prevent stroke world-wide, it is also important to spread the knowledge to all populations experiencing economic and lifestyle transitions in the developing communities so that they can avoid taking up the 'unhealthy' lifestyle and behaviours associated with modernization and westernization.

## Conclusion

This study shows a positive association between stroke prevalence and better SES using different indicators. However, compared with occupation and education, FAI may be a more sensitive and realistic index of socio-economic status for public health studies, and may inform the targeting of campaigns or other initiatives, particularly in populations where material prosperity is high in some social groups. People with higher family average income should be the priority target in the fight against stroke in China, and more attention on monitoring stroke risk factors shall be paid by themselves and health care providers.

## Abbreviations

BMI: Body Mass Index; CI: Confidence Interval; FAI: Family Average Income; GDP: Gross Domestic Products; MONICA: Multinational Monitoring of trends and determinants in Cardiovascular Disease; OR: Odds Ratio; SES: Socioeconomic Status; WHO: World Health Organization.

## Competing interests

The authors declare that they have no competing interests.

## Authors' contributions

FX contributed to study design, data collection, data analysis and paper writing. LAT participated in study design, data analysis and paper writing. XMY participated in study design, data collection, data analysis and paper writing. ITY and SG contributed to manuscript writing and language editing. All authors reviewed drafts of the manuscript and approved the final manuscript.

## Pre-publication history

The pre-publication history for this paper can be accessed here:


